# Mitochondria can orchestrate sex differences in cell fate of vascular smooth muscle cells from rats

**DOI:** 10.1186/s13293-015-0051-9

**Published:** 2015-12-16

**Authors:** E. Straface, R. Vona, I. Campesi, F. Franconi

**Affiliations:** Department of Therapeutic Research and Medicine Evaluation, Istituto Superiore di Sanità, Rome, Italy; Department of Biomedical Science, University of Sassari, Sassari, 07100 Italy; Section of Cell Aging and Gender Medicine, Department of Therapeutic Research and Medicine Evaluation, Istituto Superiore di Sanità, Viale Regina Elena 299, 00161 Rome, Italy

**Keywords:** Sex differences, Vascular smooth muscle cells, Redox balance, Cell fate, Mitochondria, Apoptosis

## Abstract

**Background:**

In basal conditions, vascular smooth muscle cells freshly isolated from aortas of male and female rats display marked sex differences in terms of redox balance and susceptibility to ultraviolet radiation-induced cell death. In particular, in the same experimental conditions, cells from male rats are more susceptible to oxidative stress and underwent apoptosis, while cells from female rats underwent premature senescence. In the present work, the mechanism involved in cell fate after ultraviolet radiation exposure is investigated.

**Methods:**

Vascular smooth muscle cells, isolated from the descending aortas of both female and male Sprague–Dawley young rats, were exposed to a single sub-cytotoxic dose of ultraviolet radiation (200 mJ/cm^2^). The distribution and the expression of molecules involved in cell survival and mitochondrial physiology were evaluated by static and flow cytometry using commercial kits and antibodies. Statistical analyses were performed by using Student’s *t* test and two-way ANOVA.

**Results:**

After exposure to ultraviolet radiation, an upregulation of survival proteins such as Bclx_L_, survivin and the presence in the nucleus of NF-κB were found in cells from females. Conversely, pro-apoptotic proteins such as Bax, caspase-3, and caspase-9 as well as loss of mitochondrial membrane potential were found in cells from male rats.

**Conclusions:**

Our results suggest that (i) mitochondria, being producers of ROS, can orchestrate sex differences in cell fate of VSMC and (ii) mitochondrial dysfunction may be a significant mechanism by which cardiovascular risk factors lead to the formation of vascular lesions in a sex-specific way.

## Background

Sex differences in the development of atherosclerosis and in particular in composition and vulnerability of plaque have been reported: females, for example, tend to have a more stable atherosclerotic plaque type than males [1]. Senescence and apoptosis of vascular smooth muscle cells (VSMC) have been shown to be important contributors to plaque vulnerability. The senescent VSMC reveal impaired plaque-repairing capacity, whereas apoptosis causes loss of cellularity in the plaque, conferring plaque vulnerability [2–5]. Reactive oxygen species (ROS) are able to trigger both phenomena. An excessive production of ROS initiates cellular damage and apoptosis [6], while a moderate production of radicals is implicated in premature senescence [7, 8].

In a previous study, we found that, in basal conditions, vascular smooth muscle cells freshly isolated from the aortas of male (MVSMC) and female (FVSMC) rats displayed marked sex differences in terms of redox balance and susceptibility to UVB-induced cell death [9]. After UVB exposure, cells from male rats underwent apoptosis, while cells from female rats have a higher propensity to undergo premature senescence [10]. Apoptosis or programmed cell death is associated with the activation of specific proteases, i.e., caspases, involving mitochondria-mediated release of apoptogenic factors. Premature senescence is associated with the appearance of several biomarkers such as β-galactosidase activity and p53 activation [11, 12]. Both apoptosis and senescence can be triggered by increased ROS levels.

Considering that the mitochondria are the main source of ROS and can orchestrate apoptosis and senescence, in this work the effects of UVB on mitochondrial physiology have been evaluated in both cell types. In particular, the mitochondrial membrane potential (MMP) increase (hyperpolarization) and the MMP loss (depolarization) were analyzed at different times after the end of UVB exposure. It is important to emphasize that the use of isolated non-tumor cultured cell, keeping a sort of sexual dimorphism, could give information on possible sex differences in mitochondrial function.

## Methods

### Cell cultures and treatments

VSMC were isolated from descending aortas of both female and male young rats. Male and female Sprague–Dawley rats (7 weeks old) were purchased from Harlan, Italy. Rats (2–3 per cage) were maintained on a 12-h light/dark cycle and allowed food and water ad libitum till the sacrifice. The experimental protocols were carried out in accordance with Italian law (DL 116, 1992) and the National Institute of Health principles of laboratory animal care (NIH 80-33, revised 1996). Primary cultures of VSMC were maintained in DMEM + GlutaMAX medium (GIBCO, Invitrogen, MI, Italy) containing 1 g/l d-glucose, supplemented with 10 % fetal calf serum, 1 % non-essential amino acids, 10 U/L penicillin, and 10 mg/ml streptomycin. Cells were seeded at density of 2 × 10^5^ cells in the Petri dishes and maintained at 37 °C. All the experiments, treatments, and corresponding controls were performed in phenol red-free DMEM culture medium (GIBCO, Invitrogen 11880-028). For fluorescence-intensified charge-coupled device video microscopy (IVM) analysis, cells were seeded on 13-mm-diameter glass coverslips in separate wells. Cells at passages 2–4 were used in this study. UVB exposure was performed as previously described [10].

### Analytical cytology

For static and flow cytometry analyses, control and treated cells were fixed with 4 % paraformaldehyde in phosphate-buffered saline (PBS) for 30 min at room temperature. After washing in the same buffer, cells were permeabilized with 0.5 % Triton X-100 (Sigma Chemical Co., St. Louis, MO, USA) in PBS for 5 min. For p66Shc, a monoclonal antibody (catalog number: 610878, BD Bioscience, Mountain View, CA) directed against this antigen was used. For Bax and Bclx_L_, polyclonal antibodies (catalog numbers: sc-493 and sc-7195, respectively, all from Santa Cruz Biotechnology, Santa Cruz, CA) directed against these antigens were used. After 30 min at 37 °C, cells were washed and then incubated with an anti-mouse fluorescein-linked or anti-rabbit fluorescein-linked whole antibodies (catalog numbers: A11008 and A11001, respectively, all from Molecular Probes, Eugene, OR, USA). To visualize distribution of mitochondria, a specific probe was used for 30 min a 37 °C (250 nM; MitoTracker Red FM, Molecular Probes, catalog number: M22425). For p65 immunostaining, cells were fixed in acetone/methanol (1:1, vol/vol) for 10 min at room temperature and air-dried. After 1 h of pre-incubation with PBS containing 10 % of AB human serum, cells were incubated with an anti-p65 antibody (catalog number: sc-372, Santa Cruz Biotechnology) for 1 h at room temperature. After three washings in PBS, cells were incubated for 30 min at room temperature with fluorescein isothiocyanate (FITC)-labeled anti-rabbit antibody (Molecular Probes). Apoptosis detection has been performed by using the following flow cytometry methods: a double staining FITC-conjugated annexin V/propidium iodide (PI) apoptosis detection kit (catalog number: BV-K101-4, MBL, Woods Hole, MA) or, alternatively, by fluorescence microscopy after cell staining with nuclear dye Hoechst 33258 (Sigma) according to the manufacturer’s protocol.

For a qualitative analysis, all samples were mounted on glass coverslips with glycerol-PBS (2:1) and observed by intensified video microscopy (IVM) with an Olympus Microphot fluorescence microscope (Olympus Corporation, Tokyo, Japan) equipped with a Zeiss CCD camera. Regarding flow cytometry analyses, all the samples were recorded with a FACScan flow cytometer (Becton–Dickinson, Mountain View, CA, USA) equipped with a 488-nm argon laser. At least 20,000 events have been acquired. The median values of fluorescence intensity histograms were used to provide a semi-quantitative analysis.

### Caspase activity

Activation state of the caspase-9 and caspase-3 was evaluated by using the CaspGLOW Fluorescein Active Caspase Staining Kit (catalog numbers: JM-K183 and JM-K183, respectively, all form MBL, Woburn, MA, USA). This kit provides a sensitive means for detecting activated caspases in living cells. The assay utilizes specific caspase inhibitors (LEHD-FMK for caspase-9 and DEVD-FMK for caspase-3) conjugated to FITC as the fluorescent marker. These inhibitors are cell permeant and non-toxic and irreversibly bind to caspase-active form. The FITC label allows detection of activated caspases in apoptotic cells directly by flow cytometry.

Cells were incubated with FITC-LEHD-FMK or FITC-DEVD-FMK for 1 h at 37 °C following manufacturer instructions. After this time samples were washed three times and immediately analyzed on a cytometer by using FL-1 channel. Samples prepared by pre-treating cells with specific inhibitors of caspase-9 or caspase-3 without FITC, also included in the kit, were considered as negative controls.

### Mitochondrial membrane potential

The MMP of control and treated cells was studied using 5-5¢,6-6¢-tetrachloro-1,1¢,3,3¢-tetraethylbenzimidazol-carbocyanine iodide (JC-1; catalog number: T-3168, Molecular Probes) as previously described [13].

### Statistical analyses

Cytofluorimetric results were statistically analyzed by using the parametric Kolmogorov–Smirnov test using Cell Quest Software. At least 20,000 events have been acquired. The median values of fluorescence intensity histograms were used to provide a semi-quantitative analysis. Results are presented as mean ± S.D of at least three independent experiments. Statistical analyses were performed by using Student’s *t* test and two-way ANOVA. A *p* ≤ 0.05 was considered statistically significant.

## Results and discussion

### Sex differences in expression of Bcl2 family proteins after UVB exposure

Recently, it has been recognized that the Bcl2 family of proteins is a critical mitochondrial intracellular checkpoint. The ratio of anti-apoptotic (Bcl2, Bclx_L_) to pro-apoptotic (Bim, Bad, Bax, Bak) molecules contributes to set the threshold of susceptibility to apoptosis.

**Fig. 1:**
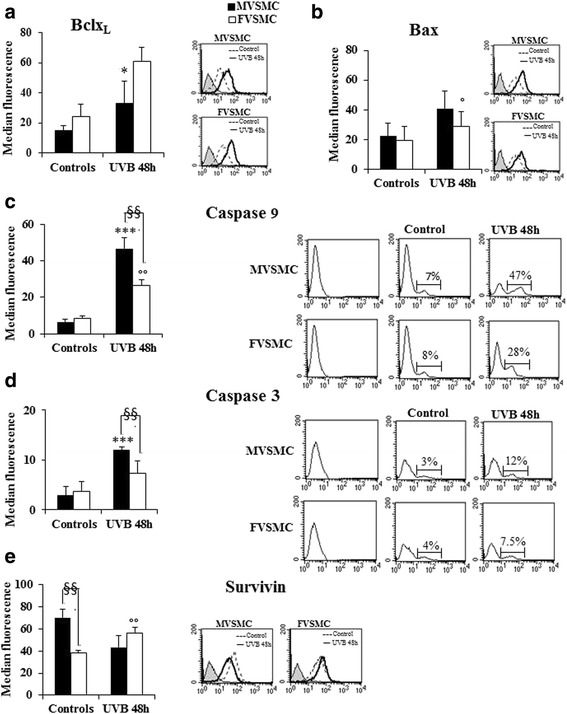
Molecules involved in cell survival. Flow cytometry analyses of ** a **Bclx_L_, **b** Bax, **c** caspase-9, **d** caspase-3, and **e** survivin 48 h in control and UVB-exposed cells. The results obtained are the mean of three independent experiments ± S.D; for each molecule, a representative experiment is shown (*right panels*). Student’s *t* test indicates (*) *p* < 0.05 MVSMC (controls vs 48-h UVB); (°) *p* < 0.05 FVSMC (controls vs 48-h UVB); (**) *p* < 0.01 MVSMC (controls vs 48-h UVB); (°°) *p* < 0.01 FVSMC (controls vs 48-h UVB); (§) *p* < 0.05 MVSMC vs FVSMC; (§§) *p* < 0.01 MVSMC vs FVSMC

Here, the expression of Bclx_L_, an anti-apoptotic protein, and Bax, a pro-apoptotic protein, was investigated in both cell types. As shown in Fig. 1a,b, at baseline, no significant sex differences were detected in the expression of these proteins. Surprisingly, 48 h after the end of UVB exposure, the expression of Bclx_L_ significantly (*p* < 0.05) increased only in FVSMC (Fig. 1a). Conversely, the expression of Bax increased significantly (*p* < 0.05) only in MVSMC (Fig. 1b). Interestingly, the increased expression of Bclx_L_ in FVSMC after UVB exposure could protect these cells from apoptosis, while the increased expression of Bax, detected in MVSMC, could give these cells an increased susceptibility to apoptosis.

### Sex differences in caspases activity after UVB exposure

It has been demonstrated that a higher expression of Bax and a lower expression of Bclx_L_ facilitate release from the mitochondria of cytochrome c (Cyt c), activating caspase-9 (initiator caspase), and then caspase-3 (effector protease) leads to apoptotic cell death. On this basis, the activity of these caspases was measured in both cell types. At baseline, no significant sex differences in both caspase-9 and caspase-3 activities were detected (Fig. 1c,d). Notably, 48 h after UVB exposure in both cell types, the activity of caspase-9 significantly increased (*p* < 0.001 for MVSMC, *p* < 0.01 for FVSMC). For caspase-3, the increase was only detected in MVSMC (*p* < 0.001). Moreover, sex differences were detected. In particular, after UVB, caspase-9 (Fig. 1c) and caspase-3 (Fig. 1d) activities were significantly higher in MVSMC than in FVSMC (*p* < 0.01 for caspase-9 and *p* < 0.05 for caspase-3).

In order to investigate if the low mortality detected in FVSMC after UVB exposure was promoted by interference of caspases with inhibitors of apoptosis protein (IAPs), the expression of survivin, the smallest member of IAP gene family, was measured by flow cytometry. Survivin is a bi-functional protein: it acts as an apoptosis suppressor and is an important factor in regulating cell division [14].

Surprisingly, sex differences were found at baseline. Survivin was significantly (*p* < 0.01) expressed in basal MVSMC than in FVSMC. After UVB exposure, survivin expression increased significantly (*p* < 0.01) only in FVSMC (Fig. 1e), and no sex differences were detected.

### Key role of NF-κB in VSMC fate

Because Bclx_L_ [15] and survivin [16] are transcriptional targets for nuclear factor kappa B (NF-κB), we assessed NF-κB activation after UVB exposure. NF-κB is a transcription factor composed of two different subunits, p50 and p65. In resting cells, these subunits are sequestered at cytoplasm level by association with the inhibitory subunit IκBα [17]. In response to oxidative stress, p65 subunit translocates to the nucleus, regulating the expression of the genes involved in cell survival [18, 19].

**Fig. 2:**
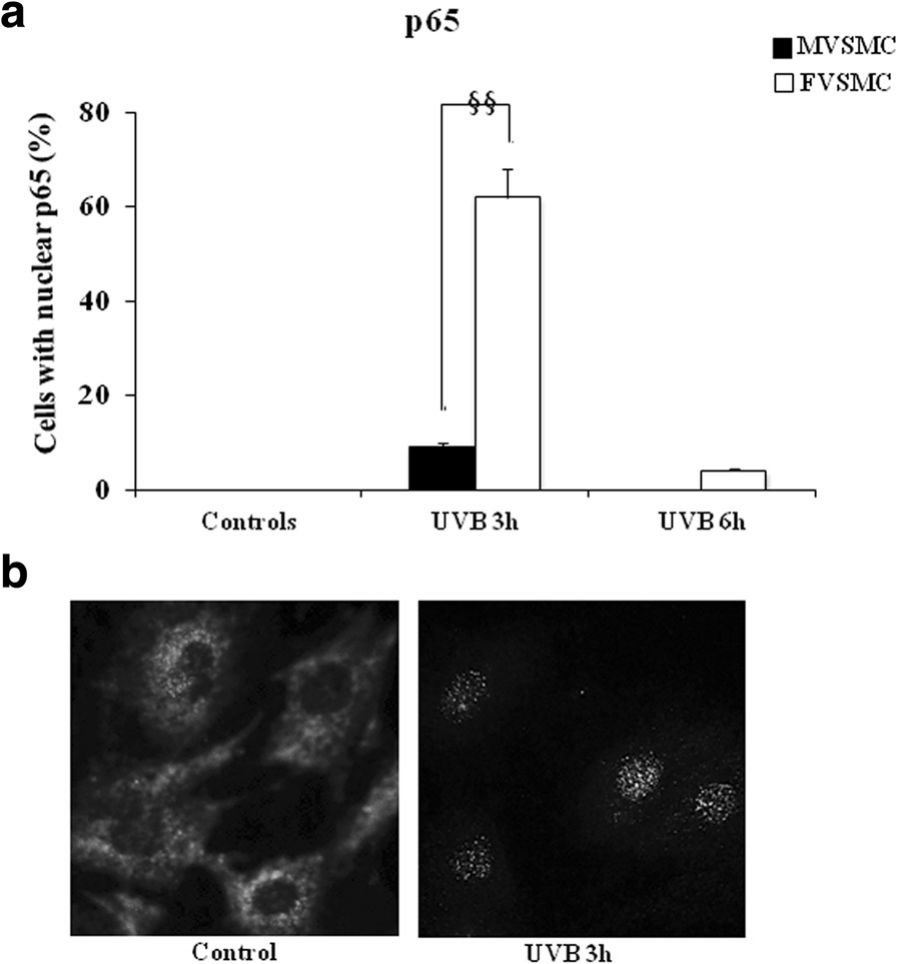
Key role of NF-κB in VSMC fate after UVB exposure. **a** Morphometric analysis of p65/subunit translocation from the cytoplasm into the nucleus. **b** Cytoplasmatic (*left panel*) and nuclear (*right panel*) p65 distribution 3 h after the end of UVB exposure. Scale bar 10 μm. (§§) *p* < 0.001 FVSMC vs MVSMC 3 h after UVB

In the present study, we investigated the role of NF-κB as a mediator of pro-survival signals in VSMC. Importantly, the morphometric analyses showed that, especially in FVSMC, the p65/nucleus association took place 3 h after UVB exposure, and it was already reduced 6 h after (Fig. 2a). Interestingly, a significant (*p* < 0.001) sex difference has been detected 3 h after the end of UVB exposure, being the percentage of cells with nuclear p65 significantly higher in FVSMC. These data suggest that, in FVSMC, the increased expression of both Bclx_L_ and survivin following UVB exposure is under the control of NF-κB. Two representative images of cytoplasmatic (left panel) and nuclear p65 distribution (right panel) are shown in Fig. 2b.

### Key role of mitochondria in stress responses of VSMC

Considering that, mitochondria play a key role in ROS production and cell death, the effects of UVB on mitochondrial physiology were evaluated in both cell types. In particular, the MMP hyperpolarization and the MMP depolarization were analyzed by flow cytometry using a JC-1 probe.

**Fig. 3:**
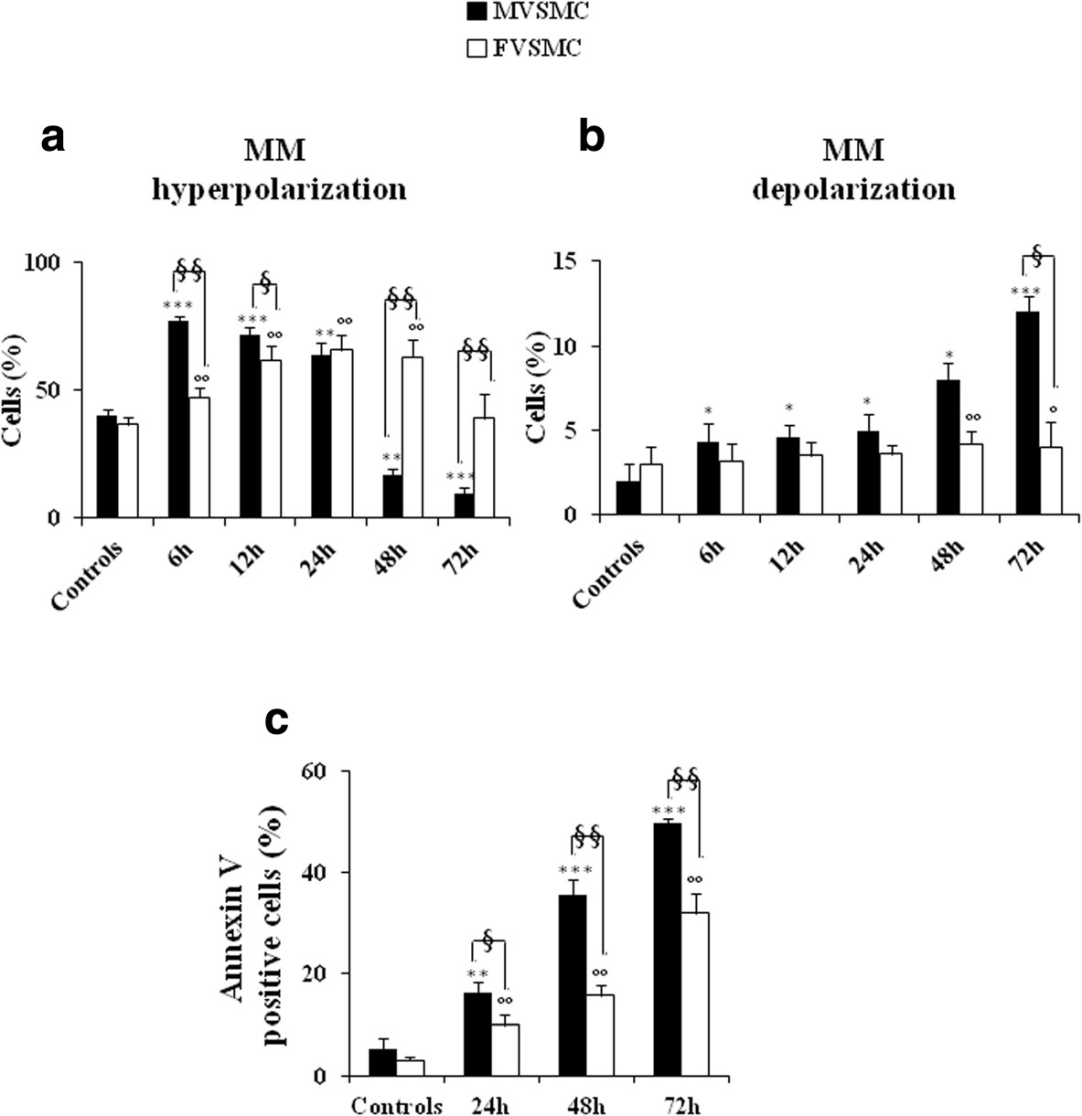
Role of mitochondria in VSMC fate after UVB exposure. Flow cytometry analyses of **a** mitochondrial membrane potential (MMP) hyperpolarization and **b** MMP depolarization at different time points starting from the end of UVB exposure. **c** Flow cytometry analyses of apoptosis after double staining with annexin V/propidium iodide at different time points after UVB exposure. The results obtained are the mean of three independent experiments ± S.D. Student’s *t* test indicates **a** (***) *p* < 0.001 for MVSMC (controls vs 6, 12, and 72 h after UVB); (**) *p* < 0.01 for MVSMC (controls vs 24- and 48-h UVB); (°°) *p* < 0.01 for FVSMC (controls vs 6-, 12-, 24-, and 48-h UVB); (§§) *p* < 0.01 MVSMC vs FVSMC 6-, 48-, and 72-h UVB; and (§) *p* < 0.05 MVSMC vs FVSMC after 12 h UVB. **b** (***) *p* < 0.001 for MVSMC (controls vs 72-h UVB); (*) *p* < 0.05 for MVSMC (controls vs 6-, 12-, 24-, and 48-h UVB); (°°) *p* < 0.01 for FVSMC (controls vs 48-h UVB); (°) *p* < 0.05 for FVSMC (controls vs 72-h UVB); (§) *p* < 0.05 MVSMC vs FVSMC 72-h UVB. **c** (***) *p* < 0.001 for MVSMC (controls vs 48- and 72-h UVB); (**) *p* < 0.01 for MVSMC (controls vs 24-h UVB); (°°) *p* < 0.01 for FVSMC (controls vs 24-, 48-, and 72-h UVB); (§§) *p* < 0.01 MVSMC vs FVSMC 48- and 72-h UVB; and (§) *p* < 0.05 MVSMC vs FVSMC after 24-h UVB

Sex differences were found in MMP polarization state after UVB exposure. Regarding MVSMC, in comparison with untreated cells, a high percentage of cells with hyperpolarized mitochondria was detected after exposure to UVB (Fig. 3a). Importantly, hyperpolarization of mitochondrial membrane (i) peaked 6 h after the end of UVB exposure (*p* < 0.0001 ); (ii) was maintained at 12 and 24 h; and (iii) significantly decreased at 48 h (*p* < 0.01) and 72 h (*p* < 0.001).

Conversely, in FVSMC, the MMP hyperpolarization induced by UVB exposure (i) started 6 h after the end of UVB; (ii) peaked from 12 to 48 h (*p* < 0.01); and (iii) decreased at 72 h, returning to the control levels (Fig. 3a).

Interestingly, we found that 48 and 72 h after the end of UVB exposure, the percentage of cells with MMP hyperpolarization in MVSMC was significantly (*p* < 0.01) lower than in FVSMC. In Fig. 3b, the percentage of cells with MMP depolarization is shown. To note that (i) in MVSMC, the percentage of cells with MMP depolarization increased significantly (*p* < 0.05) for each time analyzed and peaked 72 h after the end of UVB exposure (*p* < 0.001), and (ii) regarding FVSMC, significant differences in MMP depolarization state were detected only 48 h (*p* < 0.01) and 72 h (*p* < 0.05) after the end of UVB exposure.

A significant sex differences in MMP depolarization was detected between MVSMC and FVSMC 72 h after the end of UVB exposure (*p* < 0.05).

Importantly, we found that for both cell types, the MMP loss was accompanied by an increase of percentage of annexin V-positive cells (a marker of early apoptosis) (Fig. 3c). The highest significant (*p* < 0.001) increase in apoptotic cells was detected 72 h after the end of UVB exposure, in comparison with control cells.

Interestingly, the percentage of apoptotic cells was significantly (*p* < 0.01) higher in MVSMC than in FVSMC for each analyzed time following UVB exposure.

### p66Shc as regulator of ROS production in mitochondria

An important role in the production of ROS at the mitochondrial level is played by the protein p66Shc, a pro-apoptotic protein that acts as sensor for cellular stress, and, producing H_2_O_2_ in mitochondria, it is indispensable for mitochondrial depolarization and Cyt c release [20].

**Fig. 4:**
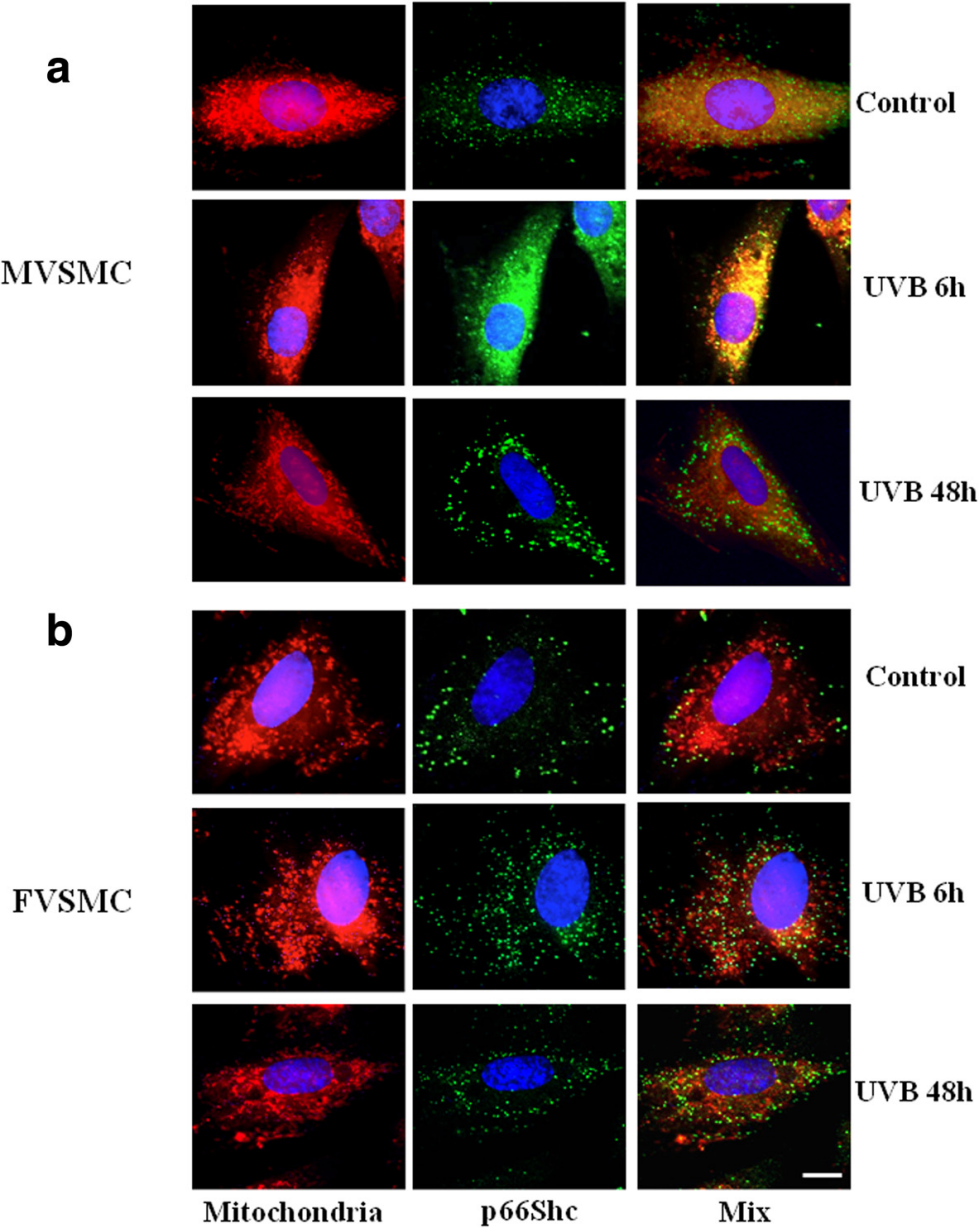
Recruitment of p66Shc from the cytosol to the mitochondria during UVB exposure. Mitochondria distribution (*red staining*, *left panels*), p66Shc localization (*green staining*, *middle panels*), and co-localization of mitochondria and p66Shc (*yellow staining*, *right panels*) in MVSMC (**a**) and FVSMC (**b**) at 6 and 48 h after the end of UVB exposure. Scale bar 10 μm

One of the mechanisms proposed is mediated by a p66Shc pool recruited from the cytosol to the mitochondria, where it acts as a redox enzyme, which produces ROS through Cyt c binding and oxidation. To investigate the possible recruitment of p66Shc from the cytosol to the mitochondria during UVB exposure, we analyzed its localization in treated cells by IVM analysis (Fig. 4). Surprisingly, a sex difference in p66Shc distribution was detected after UVB exposure. In particular, we found that 6 h after UVB exposure, p66Shc co-localized with mitochondria only in MVSMC (Fig. 4a). This co-localization was not evident 48 h after UVB exposure. Conversely, in FVSMC, UVB did not induce the recruitment of p66Shc in mitochondria (Fig. 4b). Thus, we hypothesize that in FVSMC, the absence of p66Shc in the mitochondria after UVB exposure might contribute to the protection of these cells from the apoptosis reducing ROS production and inhibiting mitochondrial depolarization.

## Conclusions

As clearly demonstrated by the results, MVSMC are more prone to apoptosis after UVB exposure. They express higher caspase-3 and caspase-9 activities and show an upregulation of the pro-apoptotic protein Bax. Moreover, MVSMC show a loss in MMP associated with a higher percentage of annexin V-positive cells. Conversely, in FVSMC, a positive regulation of the molecules involved in cell survival, with an anti-apoptotic effect, has been observed after UVB exposure (lower caspases activity, higher survivin levels, and higher percentage of cells with nuclear p65). Furthermore, the loss in MMP is associated with a lower increase in the percentage of annexin V-positive cells.

Taken together, the results suggest that mitochondria, being producers of ROS, can orchestrate sex differences in cell fate of VSMC. Moreover, as described in literature [21, 22], mitochondrial dysfunction may be a significant mechanism by which cardiovascular risk factors lead to the formation of vascular lesions. Therefore, the sex differences reported in the present work could help to better understand the origin of sex differences observed in cardiovascular risk profile including those reported for atherosclerosis [23, 24].

Importantly, this research also suggests that (i) it is essential to indicate the sex of cells and plan experiments on male and female cells, to understand the basics of the observed differences in the male and female pathophysiology, and (ii) the idea that isolated cells could maintain a sort of “memory” of their origin, i.e., a sexual dimorphism, could provide new insight in understanding pathogenesis and outcome of some diseases, including cardiovascular diseases.

## Abbreviations

Cyt c: cytochrome c
FVSMC: vascular smooth muscle cells isolated from female rats
IAPs: inhibitors of apoptosis protein
MMP: mitochondrial membrane potential
MVSMC: vascular smooth muscle cells isolated from male rats
NF-κB: nuclear factor kappa B
PBS: phosphate-buffered saline
ROS: reactive oxygen species
VSMC: vascular smooth muscle cells
